# Nitrous Oxide in Labor

**Published:** 1883-08

**Authors:** 


### NITROUS OXIDE IN LABOR.

Dr. Tittel gives a report (*Wiener Med. Blatter*) of over fifty trials which he has made of the inhalation of nitrous oxide gas in parturition. He employed it chiefly in primiparæ with very severe pains, and found a diminution of the suffering in every case. He found that it acted better when given in the first stage, as its effects lasted into the second, and quiet inhalation was more difficult when it was attempted to be given in the second stage. The pulse was generally retarded, and the fœtal pulsations on the contrary, generally accelerated. The pains were, in many instances, increased in strength and frequency, and Dr. Tittel found this action of the gas very serviceable in multiparæ with few and weak pains. Vomiting was arrested in four cases by the inhalation of gas, and the only evil results which were observed were two cases of convulsions, one hysterical, and the other true epileptic.—*Medical Record.*

